# Cognitive Bias Under Adverse and Rewarding Conditions: A Systematic Review of Rodent Studies

**DOI:** 10.3389/fnbeh.2020.00014

**Published:** 2020-02-12

**Authors:** Ho A. T. Nguyen, Chao Guo, Judith R. Homberg

**Affiliations:** Donders Institute for Brain, Cognition, and Behavior, Radboud University Medical Center, Nijmegen, Netherlands

**Keywords:** cognitive bias, rodents, stress, reward, generalization, ambiguous cues

## Abstract

**Background:** Cognitive bias refers to emotional influences on cognition and provides a cognitive measure of negativity- or positivity-bias through assessment of the behavioral responses to ambiguous stimuli. Thus, under negative conditions an animal is more likely to judge ambiguous stimuli as negative, and under positive conditions as positive. The transfer of past experiences to novel but similar situations is highly adaptive, as it allows the animal to anticipate on the most likely outcome of the ambiguous cues.

**Methods:** We conducted a systematic review to summarize the current state of evidence on cognitive bias in rodents under adverse and rewarding or supportive conditions.

**Results:** In total 20 studies were identified, in which auditory, spatial, tactile, or visual tasks were used. Stressed rodents generally made fewer positive responses than their non-stressed conspecifics. Housing enrichment made rodents more positive in anticipation of ambiguous cues. Ethanol seeking rats generalized the ambiguous cues to sucrose and less to ethanol if sucrose was available. Amphetamine, fluoxetine, and ketamine shifted the bias toward positivity, while reboxetine elevated negative bias.

**Conclusion:** The auditory tasks have been most extensively validated, followed by the tactile and spatial tasks, and finally the visual tasks. The tactile and spatial tasks use latency as readout, which is sensitive to confounding factors. It is yet uncertain whether spatial tasks measure cognitive bias. Across all tasks, with some exceptions, rodents exposed to stress show less positivity-bias when exposed to ambiguous cues, whereas rodents exposed to rewarding substances or treated with antidepressant drugs are biased toward reward. Considering the methodological heterogeneity and risk of bias, the present data should be interpreted with caution.

## Introduction

### Rationale

Cognitive bias focuses on the behavior elicited by ambiguous cues that are intermediates of two types of cues associated with punishment or reward (Enkel et al., 2010). Cognitive bias is therefore crucial for fast and efficient adaption to the environment that can be either dangerous or rewarding (Enkel et al., 2010; Norbury et al., 2018). Furthermore, as cognitive bias is strongly influenced by emotional state across species from birds to rodents to primates (Roelofs et al., 2016), the assessment of cognitive bias provides a readout of emotion and well-being with high translational value. An important component of cognitive bias is conditioning. Conditioning is the process through which behavior is associated with an unconditioned motivationally relevant stimulus. Such a stimulus can trigger approach or avoidance behavior. Rodents can store conditioned stimuli in their working memory and assess stimulus differences and similarities (Fassihi et al., 2014). When stimuli are ambiguous, generalization can occur. Generalization refers to the process of adaption to novel, but comparable situations or objects (ambiguous cue) by transferring stored information inferred from learned behavior to the present situation (Norbury et al., 2018).

In humans, cognitive biases can come in three flavors, namely attentional bias (e.g., speeded reaction times toward negative or positive stimuli vs. neutral stimuli), memory (e.g., enhanced recall of negative or positive words), and interpretation bias (e.g., resolution of ambiguous cues in a negative or positive way) (Platt et al., 2017). There is the general hypothesis that negative conditions lead to negative cognitive biases, and that positive conditions lead to positive cognitive biases. In support, individuals suffering from major depression show a negative interpretation bias (Everaert et al., 2017), for instance when they have to form a sentence from a set of words and write it down. This results in more negatively framed sentences among the alternative sentences that could be formed compared to those produced by healthy subjects. This is not only seen in depressed individuals, but also individuals who are at risk for developing depression (Sfarlea et al., 2019). While studies on interpretation bias in substance use disorder patients are lacking, it has been demonstrated that cannabis, opioid, and stimulant dependent subjects show a positive cognitive attention bias (Zhang et al., 2018). For instance, in a dot probe task, in which participants are presented with images including drug-related pictures, drug dependent individuals display a significant shorter reaction time to a dot that is presented at the location were the drug-related pictures were previously shown compared to non-drug related pictures (Frankland et al., 2016).

### Objective

Cognitive bias is seen across species, indicating that it has an important role in daily life functioning. Here, we focus on interpretation bias, because it relies on the assignment of valence to neutral stimuli, a cognitive ability that is common across species. Rodents, which are widely used as a disease model, have significantly contributed to emotion-related research (Malakoff, 2000). Rodent models of cognitive interpretation bias (hereafter referred to as cognitive bias) are emerging, but different types of tasks have been developed. This raises the question as to whether the effects of negative and positive events on generalization bias are comparable across tasks. To assess the current state of evidence in rodent studies, we conducted a systematic review. To the best of our knowledge, a systematic review on cognitive bias tasks does not exist yet for rodents. For narrative reviews that more deeply explain various principles of cognitive bias measurements in rodents we refer to Hales et al. (2014), Roelofs et al. (2016).

## Materials and Methods

### Systematic Review Protocol and Search Strategy

A systematic search strategy was developed. Published papers were identified in PubMed in cooperation with a methodologist in literature mining of the medical and science library. MeSH terms for depression, addiction, generalization, and (cognitive) bias were used (Table S1). All rodent studies were detected by the animal search filter developed for PubMed (Hooijmans et al., 2010).

The search was performed on 19 December 2018. The references were imported to Early Review Organizing Software (EROS; Institute of Clinical Effectiveness and Health Policy, Buenos Aires, Argentina). In the first stage of screening, papers about the effect of depression and addiction on cognitive bias and generalization were screened based on title and abstract. There was exclusive reliance on English language studies. Studies were excluded if they were: (1) duplicated, (2) not a behavioral study, (3) not about depression or addiction, (4) included co-morbidities other than depression and addiction, (5) not about generalization or cognitive bias, and/or (6) not a rodent study. The articles were then sorted out for reviews that were used to find additional publications. Two reviewers independently performed the screening on title and abstract according to the selection criteria. If disagreement appeared, decisions were made by informal consensus. During the screening phase, included articles found by the systematic search and known articles were screened on full text with the same criteria. Full- text screening and further assessment were conducted by one reviewer.

### Study Characteristics and Data Extraction

Data were extracted to give a comprehensive overview of all included studies (Table S2). Items include information about the rodent (number of animals, species, strain, gender, age, body mass, intervention, testing frequency), the type of behavioral task, nature of stimuli (e.g., visual, auditory), and stimulus presentation procedure (length and frequency stimuli exposure, nature of reference and ambiguous cues). It was checked if reasons for fallouts of animals were reported adequately. Author and year of publication are listed. Any outcome measure was included if clearly showing the incidences of cognitive bias with a measurable unit. Authors were not contacted in case of unclear reporting.

### Assessment Risk of Bias

The risk of bias of the included studies was assessed according to Hooijmans et al. (2014), with slight modifications (the number of criteria was reduced to the most critical 10 criteria applicable to the current papers selected). An adapted bias tool was used to give an overview of the risk of bias of all included studies. Items assessed are: (1) Study objective is described, (2) Main outcomes are described, (3) Rodent characteristics are clear, (4) Experimental task is described, (5) Outcomes are valid and reliable (criteria 1, 2, 3, 4, 6, 7, 8, 9 are marked as sufficient), (6) Cofounders are described and corrected (counterbalancing was set as criterion), (7) Statistics was used properly [data tested for normal distribution, transformation applied when necessary, proper application of parametric or non-parametric tests, p-hacking made clear (number of dependent variables, number of tests, exclusion of animals/outliers)], (8) Power-analysis is provided, (9) Main findings are reported, (10) Dropouts are described and explained. Three different scores were used (yes: low risk of bias, insufficient: high risk of bias, unclear: unknown risk of bias) (Table S3). Authors were not contacted in case of unclear reporting.

### Comparison of Studies

Since no meta-analysis was performed and authors were not contacted in case of unclear reports, we analyzed the data by clustering the comparisons of the studies by their behavioral task. Results were compared if possible. By doing so, we increased the methodological similarity and decreased heterogeneity of study results among included comparisons. We assessed whether contradictions or similarities were reported. Finally, a descriptive synthesis of the different subgroups for an overall estimation was performed if feasible.

## Results

### Literature Search and Study Characteristics

**Figure 1:**
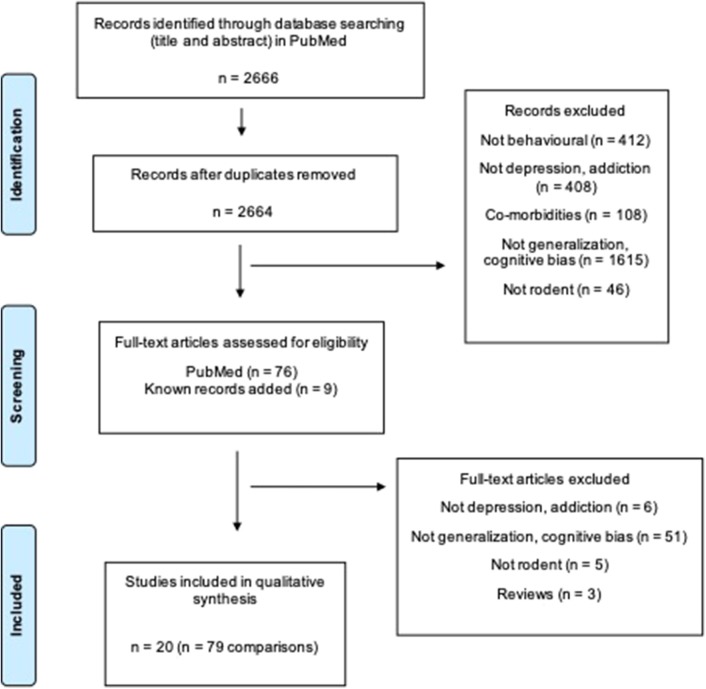
Flow diagram of the systematic review. Two thousand six hundred sixty-six articles are found. Twenty studies including 79 comparisons are included in the final review. Two groups (e.g., one control and one treatment group) are defined as a comparison (Moher et al., 2009).

The search strategy (Table S1) yielded 2,664 unique papers in PubMed. Twenty studies compromising 79 comparisons investigated generalization bias in rodents (Figure 1).

Studies used different methods (Table S2, Part 1, 2). Male rodents are dominating in 82% of the comparisons, while females are participating in 14% of the comparisons. In 4% of the comparisons, unclear indications are made. Rats are most commonly used (82%), whereas mice appear in 15% of the comparisons and hamsters in 2.5% of the comparisons.

Among all comparisons, different interventions or strains have been used to manipulate the emotional state of the laboratory rodents. Two comparisons (one study) use serotonin transporter (5-HTT) knockout mice (Kloke et al., 2014). Six comparisons (four studies) introduced stress chronically (Chaby et al., 2013; Papciak et al., 2013; Rygula et al., 2013; Novak et al., 2016). In two comparisons (two studies), enrichment of housing was removed (Burman et al., 2008; Bethell and Koyama, 2015). One comparison randomly intervened with the housing of rats using different treatments (Harding et al., 2004) that may be comparable to the interventions in rats where stress was enforced over a longer period (Chaby et al., 2013; Papciak et al., 2013; Rygula et al., 2013; Novak et al., 2016). Drug interventions were given to rats in 39 comparisons (Enkel et al., 2010; Anderson et al., 2013; Hales et al., 2017; Drozd et al., 2018).

The comparisons can be divided into three subgroups according to their behavioral task. Six studies that included 17 comparisons use spatial orientation of the cues (Burman et al., 2008, 2009; Richter et al., 2012; Kloke et al., 2014; Bethell and Koyama, 2015; Krakenberg et al., 2019). Ten studies including 55 comparisons use auditory cues (Harding et al., 2004; Enkel et al., 2010; Anderson et al., 2013; Ginsburg and Lamb, 2013; Papciak et al., 2013; Rygula et al., 2013; Rygula and Popik, 2016; Drozd et al., 2017, 2018; Hales et al., 2017). Four studies including six comparisons rely mainly on tactile cues (Brydges et al., 2011, 2012; Chaby et al., 2013; Novak et al., 2016). One study including one comparison implemented visual cues (Krakenberg et al., 2019).

### Risk of Bias in Prevalent Studies

**Figure 2:**
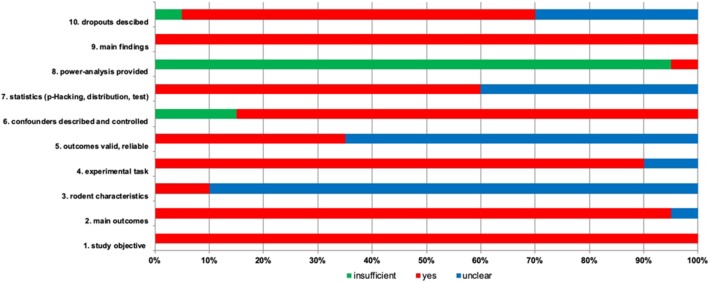
Risk of bias is assessed for included studies with 10 items. The percentage for each item is given with three options. Green, insufficient; red, yes; blue, unclear reporting.

Most articles reported main outcomes (95%) (Figure 2) (Harding et al., 2004; Burman et al., 2008, 2009; Enkel et al., 2010; Brydges et al., 2011, 2012; Richter et al., 2012; Anderson et al., 2013; Chaby et al., 2013; Ginsburg and Lamb, 2013; Papciak et al., 2013; Rygula et al., 2013; Kloke et al., 2014; Bethell and Koyama, 2015; Novak et al., 2016; Rygula and Popik, 2016; Drozd et al., 2017; Hales et al., 2017; Krakenberg et al., 2019). Rodent characteristics were missing and unclear in 90% of the papers, while sufficient report was presented in 10% of the papers (Anderson et al., 2013; Krakenberg et al., 2019). The experimental task was clear in 90% of the papers, but in two studies reporting was unclear (5%) (Novak et al., 2016; Drozd et al., 2018). 35% of the studies provided reliable outcomes (Richter et al., 2012; Chaby et al., 2013; Ginsburg and Lamb, 2013; Rygula et al., 2013; Kloke et al., 2014; Rygula and Popik, 2016; Krakenberg et al., 2019). Confounders have been described appropriately (85%) and insufficiently (15%) (Harding et al., 2004; Burman et al., 2008, 2009). Statistics was conducted adequately (60%) or remains unsure (40%) (Harding et al., 2004; Burman et al., 2008; Enkel et al., 2010; Anderson et al., 2013; Papciak et al., 2013; Drozd et al., 2017, 2018; Hales et al., 2017). Only one study provided a power analysis (5%) (Drozd et al., 2018). All studies described the main findings (100%). Animal dropout has been reported in 65% of the studies, while in 30% of the studies reporting is unclear (Harding et al., 2004; Enkel et al., 2010; Brydges et al., 2011; Anderson et al., 2013; Ginsburg and Lamb, 2013; Novak et al., 2016). As sum of the found biases, it was unclear (due to limited information on the experimental design and data analysis) whether the outcomes were valid and reliable (65%) (Harding et al., 2004; Burman et al., 2008, 2009; Enkel et al., 2010; Brydges et al., 2011, 2012; Anderson et al., 2013; Papciak et al., 2013; Bethell and Koyama, 2015; Novak et al., 2016; Drozd et al., 2017, 2018; Hales et al., 2017).

## Comparison of Studies

### Auditory Task

#### Cognitive Bias Under Negative Conditions

##### No treatment intervention

In depression-related research it is assessed whether negativity-biased animals are biased toward tones predicting punishment. Healthy negativity-biased animals expect punishment when confronted with ambiguous tones. Most of the auditory studies are derived from the same research groups (Papciak et al., 2013; Rygula et al., 2013; Rygula and Popik, 2016; Drozd et al., 2017, 2018) and are referring to the methods of Enkel et al. (2010). The general procedure is similar for all studies using the auditory paradigm. The generalization bias test is preceded by a discrimination training of two different tones (high and low frequency) signaling the delivery of a sucrose pellet or an electric footshock. Animals have to press one of two levers, which are associated to one of the two tones, to either avoid the electric shock or to receive a sucrose pellet. The cognitive bias test consists of trials presenting ambiguous frequencies in between the high and low learned reference frequencies. After rats have been tested in a first session, several studies proceeded to categorize the rats into negativity-biased or positivity-biased rats based on a bias index (Papciak et al., 2013; Rygula et al., 2013; Rygula and Popik, 2016; Drozd et al., 2017, 2018). It is found that negativity-biased animals make less positive lever presses and more negative lever responses in anticipation of the ambiguous sound (Papciak et al., 2013; Rygula et al., 2013; Rygula and Popik, 2016; Drozd et al., 2017) and the negative tone (Rygula et al., 2013) than positivity-biased rats. Furthermore, healthy negativity-biased animals make more correct choices than positivity-biased animals when confronted with tones signaling predominantly punishment and reward.

To assess sensitivity to negative and positive feedback in negativity-biased and positivity-biased rats the probabilistic reversal learning task (PRL) has been used. In this task animals are trained to lever press. Correct lever pressing predicts sucrose in 80% of the trials and electric shock in 20% of the trials. The incorrect lever predicts electric shock in 80% of the trials and sucrose in 20% of the trials. After eight consecutive correct lever presses, the correct lever becomes the incorrect lever and vice versa (Rygula and Popik, 2016). It has been found that negativity-biased rats respond more often correctly than positivity-biased rats in the PRL task, because they more frequently change their response after being punished for making an incorrect choice if the lever predicted predominantly punishment. Negativity-biased rats, therefore, differentiate between the lever signaling punishment and the lever signaling reward to avoid electric shocks more often. It is possible that the negativity-biased rats make more correct decisions because they change their response upon true punishment more compared to the positivity-biased rats. Alternatively, negativity-biased rats may take less risk than positivity-biased rats. Rats taking less risk are more precise in following the rules and they can “calculate” probabilistic chances of reward and punishment. The latter hypothesis better explains why the general PRL performance does not differ significantly between positivity-biased and negativity-biased groups (Rygula and Popik, 2016).

##### Physical stress treatment

It is assumed that stressed animals are less motivated to gain reward and expect more punishment when hearing ambiguous tones. Animals undergoing physical treatment to introduce stress make more negative responses than positive responses using the same task as described previously in healthy rats. It has been demonstrated that stressed negativity-biased animals are taking more time to respond than non-stressed positivity-biased and negativity-biased rats, implying that stressed negativity-biased rats have less motivation to approach stimuli linked to sucrose (Drozd et al., 2017). Tests on anhedonia that were conducted next to the cognitive bias tests show that chronic stress diminishes sucrose intake in negativity-biased and positivity-biased rats (Rygula et al., 2013). Anhedonia was observed in helpless rats as well (Enkel et al., 2010). Reduced sucrose preference was more prominent and prolonged in negativity-biased rats (Papciak et al., 2013). It has also been confirmed that rats socially defeated made more negative and less positive lever responses than non-stressed rats upon presentation of the ambiguous tone. Subjects were more negative after stress and more negative than undefeated rats (Papciak et al., 2013). Furthermore, rats living under unpredictable housing conditions were slower in making positive responses and tended to respond less to food tone and near-positive tone (Harding et al., 2004). Similarly, helpless rats less often pressed the positive lever in anticipation of the mid and near negative tone. They more frequently pressed the negative lever in response to the mid tone (Enkel et al., 2010). The overall response preference was more negative in helpless than in non-helpless subjects as it is detected in untreated negativity-biased rats (Enkel et al., 2010; Papciak et al., 2013; Rygula et al., 2013; Rygula and Popik, 2016; Drozd et al., 2017).

Several studies assume that animals are inherently positively or negatively biased toward the auditory cues since the reference tones are believed to be “reward/positive” or “punishment/negative” (Papciak et al., 2013; Rygula et al., 2013; Rygula and Popik, 2016; Drozd et al., 2017, 2018). It is assumed that there is no neutral behavior or co-occurrence of negative and positive bias within an individual's decision-making process. There is, however, a fluctuation within a group implying that cognitive biases alter across time (Rygula et al., 2013; Rygula and Popik, 2016). Animals shift between positive or negative bias across different days without intervention. Although a significant difference between positivity-biased and negativity-biased groups remained, future studies need to investigate fluctuations within a subject. This should be investigated without having predefined rats based on their cognitive bias score to avoid treatment and analysis bias.

##### Antidepressant treatment

Because depression is associated with negativity-bias and antidepressants decrease (in a certain subject of individuals) negativity-bias (Warren et al., 2015), it is likely that they contribute to a shift toward positivity-bias. A few studies tested the effects of antidepressant drugs on cognitive bias (Enkel et al., 2010; Anderson et al., 2013; Hales et al., 2017). In these studies, two reference tones again predicted punishment or reward. Rats had to press one of two levers, which linked to one of the tones, to either avoid punishment or receive reward. Cognitive bias was subsequently tested with ambiguous tones (Enkel et al., 2010; Anderson et al., 2013). Upon co-administration of reboxetine (a norepinephrine reuptake inhibitor) and corticosterone, rats showed less positive lever presses for all tones compared to controls. More omissions of positive, near positive and mid tones occurred (Enkel et al., 2010). Reboxetine treatment replicated the response rate of positive lever presses upon ambiguous tone presentation of helpless rats (Enkel et al., 2010). In contrast, Anderson et al. (2013) could not find a negative bias in reboxetine treated rats (Anderson et al., 2013). There was, however, a trend to an enhanced negative bias in anticipation of the midpoint tone. A decrease in reward lever presses and an increase in omissions was observed. Rats treated with reboxetine spent more time in anticipation of the positive tone (Anderson et al., 2013). Hales et al. (2017) trained rats to discriminate two tones predicting sugar reward, one with a higher and the other with a lower sugar value. They found that reboxetine also tended to enhance negative bias (Hales et al., 2017). In contrast to acute intraperitoneal reboxetine injection, 3 weeks of subcutaneous fluoxetine injection led to a shift toward positive bias upon presentation of the ambiguous tone during the time of treatment (Hales et al., 2017). Summarized, both helpless rats and reboxetine treated rats are negatively biased (Enkel et al., 2010), whereas chronic fluoxetine treatment contributes to a positive bias (Hales et al., 2017). Antidepressant treatment thus does not necessarily induce a shift toward positivity bias in animals. The inability of reboxetine to induce positivity-bias seems to contradict antidepressant effects on cognitive bias in humans (Warren et al., 2015), asking for further tests to assess the predictive validity of auditory tasks.

#### Cognitive Bias Under Positive Conditions

It is hypothesized that exposure to rewarding stimuli shifts bias to a positive interpretation of ambiguous cues. This is because repeated experience with reward becomes tied to the prediction that future outcomes are also rewarding (Alessandri et al., 2008). Hales et al. (2017) trained rats to discriminate two tones predicting sugar reward with high or low sugar value by pressing one of two levers before generalization bias was tested with an ambiguous tone (Hales et al., 2017). Rats, additionally, were treated with amphetamine, ketamine and cocaine. The researchers found that amphetamine and ketamine induced a positive bias upon presentation of the ambiguous tone.

Some studies used multiple ambiguous tones (Harding et al., 2004; Enkel et al., 2010; Anderson et al., 2013), while other studies used one ambiguous tone (Papciak et al., 2013; Rygula et al., 2013; Rygula and Popik, 2016; Drozd et al., 2017). An advantage of using multiple ambiguous tones is that a response curve can be generated illustrating the extent to which generalization occurs according to its steep and asymptote. Thereby, the width of the generalization curve can be measured. Ginsburg and Lamb (2013) developed an ethanol seeking model to assess the change of cognitive bias in rats and derived a function from the curve (Ginsburg and Lamb, 2013). More specifically, Ginsburg and Lamb (2013) trained rats to discriminate between two tones (8, 16 kHz), both of which predicted ethanol and sucrose by pressing one of two available levers (Ginsburg and Lamb, 2013). One lever was linked to ethanol and the other lever was linked to sucrose delivery. Rats were rewarded with sucrose or ethanol if one of the levers was pressed five times upon presentation of 8 kHz. Sixteen kilohertz predicted sucrose if the corresponding lever was pressed 150 times or ethanol if the other lever was pressed five times. Rats were, subsequently, confronted with multiple ambiguous tones (6, 10, 12, 14, 18 kHz) to test generalization. During discrimination, rats more often responded to the sucrose associated lever when 8 kHz was presented, while 16 kHz led to more ethanol lever presses. This response behavior remained even after rats were conditioned to link 8 kHz and 16 kHz with a higher fixed ratio to gain sucrose and a lower fixed ratio to gain ethanol. Rats, therefore, discriminate the tones and link them to a specific reward. It is possible that this discrimination is necessary for “orientation” to predict sucrose and ethanol availability. Upon presentation of the ambiguous cues, rats generalized the ambiguous tones less to ethanol, but more to sucrose after the 8 kHz trial had been presented several times at the beginning of the session. Ethanol withdrawal did not change the response curve and sucrose was preferred when the ambiguous tones were presented. These results indicate that the presence of a second reward, here sucrose, reduces ethanol consumption, even after ethanol withdrawal. However, whether sucrose is truly more positive than ethanol cannot be derived from this study. In summary, amphetamine and ketamine induce a positive bias in rats. Furthermore, sucrose can diminish the bias toward alcohol by shifting the positive bias of reward to sucrose.

## Spatial Task

### Cognitive Bias Under Baseline, Negative, and Positive Conditions

In the spatial paradigm two locations are provided that are separated from each other's by distance. One location contains a stimulus that is positive or preferred by the animal and the other location contains a stimulus that is negative or less preferred by the animal. The ambiguous cue is an unknown location that is located in between these two reference locations, so that generalization bias is assessed based on the spatial distance. Like in the auditory paradigm, some studies used one, while others used more than one ambiguous cue, here referred to as the ambiguous location.

Bethell and Koyama (2015) manipulated the emotional state of hamsters by adding or removing housing objects (Bethell and Koyama, 2015). The hamsters were confronted with the two reference spouts located on the opposite sides of one wall in an arena. One spout was conditioned to sucrose and the other spout to quinine hydrochloride, which is aversive to hamsters. The cognitive bias test consisted of the same setting with three more spouts located near the sucrose, near the quinine hydrochloride and one spout located at the midpoint between the reference spouts. Hamsters in enriched housing approached the two reference spouts associated with sucrose or quinine hydrochloride more frequently than hamsters in which enrichment was removed. There was, nevertheless, no difference in approaching the ambiguous locations between enrichment and removed enrichment group. It was, moreover, not entirely clear whether there was a difference within the group before adding (or after removing) enrichment.

Richter et al. (2012) tested rats in a radial maze with five arms (Richter et al., 2012). During testing, three ambiguous arms located between the two outer arms, which were separated by a distance of 180° from each other, were accessible. The positive arm contained a fruit-loop, while the negative arm contained a quinine fruit-loop. Congenitally helpless and non-helpless rats were compared and housing conditions were altered. It was found that helpless rats spent less time to reach the pots of the negative and ambiguous locations than non-helpless rats, but housing enrichment did not have an influence on generalization and latencies between groups. There was still a greater difference in latencies between the negative arm and ambiguous arms in non-helpless than in helpless rats. The results imply that helpless rats are impulsive or hyperactive and that helpless rats tend to generalize the outcome of the ambiguous arms to the negative arm.

Kloke et al. (2014) used wild-type mice and serotonin transporter (5-HTT) knockout mice, which display increased anxiety, and in the presence of stress elevated levels of depression-like behavior (Homberg and van den Hove, 2012). The researchers investigated whether 5-HTT knockout mice were cognitively biased toward negativity using a radial maze with multiple arms in two independent experiments (Kloke et al., 2014). In one experiment, it was assessed whether 5-HTT knockout in mice had an influence on reaching the end of an ambiguous location in a maze with three arms from which two arms were the negative (air-puff) and positive (access to home-cage) arm and the third arm was located centrally functioning as ambiguous location. 5-HTT knockout mice were slower to reach the end of the central arm than controls, but this was not significantly different between the two genotype groups. This indicates that lacking 5-HTT does not influence cognitive bias of spatial location in mice. In another experiment, the researchers investigated whether wild-type mice were cognitively biased using a maze with five arms from which two arms were the negative (air-puff) and positive (access to home-cage) arm. Three arms functioned as ambiguous arms. In this experiment, animals were divided into three groups, so that one of the three ambiguous arms were accessible to one group (near-positive, near-negative, central arm). Mice reached the near positive-arm faster than the central and near-negative-arm, indicating that wild-type mice are positively biased to the near-positive location. Mice also reached the end of the central arm faster and spent more time at the central arm than in the near-negative arm, supporting that the near-negative location is generalized to the negative arm. A limitation of this experiment was that wild-type mice had no access to all three ambiguous locations at the same time. Mice showed individual fluctuations in the latency to reaching the positive and negative arm. It is, thus, possible that the latencies of reaching the arms between the three groups are over- or underestimated (Kloke et al., 2014).

Burman et al. (2008) performed experiments with an unrewarded and rewarded pot and three ambiguous pots in between in a radial arena with rats (Burman et al., 2008). The latency to reach the near-unrewarded pot was longer in the rats living under unenriched housing conditions than rats of enriched housing. No significant difference of reaching the ambiguous pots between the groups was detected. It is worth mentioning that the researchers filtered the rats based on the latency time of the reference cues. Animals that spent less time on arriving to the negative pot than the positive pot were excluded from the study. Furthermore, animals that spent less time on reaching the location/pot associated with “no-reward/negative stimuli” than the location linked to “reward/positive stimuli” were not included. It is, therefore, not surprising that the time to reach the rewarding pot significantly differed from the time to approach the unrewarded pot. If the animals that spent less time on reaching the “negative/unrewarded” location would have been included, studies could have still assessed whether animals carry a cognitive bias despite their preference toward the “negative/unrewarded” location and avoidance toward the “positive/rewarded” location. The cognitive bias of animals preferring the “negative/unrewarded” location could be compared to the cognitive bias of animals preferring the “positive/rewarded” location. It could have been hypothesized that cognitive bias occurs in both groups, but in opposite directions so that the ambiguous cues are generalized to the preferred location.

In another study Burman et al. (2009) exposed rats to a central illuminated arena with high or low intensity in a radial maze containing five arms (Burman et al., 2009). One arm was associated with reward containing food pellets and the aversive arm, that was located 180° away from the reward arm, contained a quinone-soaked pellet. The three arms in between were the ambiguous arms. No treatment difference between high and low illumination in latencies to reach the pots of the arms was observed. Rats reached the doors of the three ambiguous arms in the same time range emphasizing that there was no generalization of the locations.

In the study of Krakenberg et al. (2019), mice entered a tunnel with a specific length connected to a goal-box with two pots located at either the left or right side (Krakenberg et al., 2019). Mice associated a 50 cm tunnel with a large almond piece on the left side of the goal-box (positive tunnel), while a 10 cm tunnel was linked to a small piece of almond on the right side (negative tunnel). During the test, one of three available ambiguous tunnels of different lengths (20, 30, or 40 cm) were entered by the mice and bias was tested according to their choice. Mice were positivity-biased when they entered the near-positive tunnel and negativity-biased when they entered the near-negative tunnel, while there was no bias when mice crossed the central tunnel. This paradigm shows that using different tunnel lengths can be used as the cues to test cognitive bias in mice.

In summary, current evidence on cognitive bias using spatial tasks is contradictory since rodents are not biased upon presentation of the ambiguous locations. They have learned to associate one reference location with a negative and the other location with a positive outcome in some studies (Burman et al., 2008, 2009; Richter et al., 2012; Bethell and Koyama, 2015), but it is not clear whether mice are truly positivity-biased when the ambiguous location is located close to the positive location (Kloke et al., 2014). Only one study showed that mice were positivity-biased when the ambiguous location was comparable to the positive location (Krakenberg et al., 2019). Moreover, helpless rats tended to generalize ambiguous locations to the negative location (Richter et al., 2012). Furthermore, housing enrichment did not alter cognitive bias when animals were exposed to enriched housing conditions as compared to rats that has been exposed to unenriched housing (Burman et al., 2008; Richter et al., 2012; Bethell and Koyama, 2015), but rats took less time to check the objects of the ambiguous locations after their standard housing was changed to enriched housing. This emphasizes that housing conditions affect the state of mood (Richter et al., 2012).

## Tactile

### Cognitive Bias Under Baseline, Negative, and Positive Conditions

Among the studies employing a tactile paradigm the study of Novak et al. (2016) was excluded since the experimental task was unclear and results were therefore unreliable (Novak et al., 2016). The general procedure of the remaining studies was comparable (Brydges et al., 2011, 2012; Chaby et al., 2013). Rats had to cross a tunnel connected to a goal-box signaling a reward on the left or right side of the goal-box. The walls of the tunnels were covered with a specific sandpaper predicting the location of the reward. The coarse-sandpaper covered tunnel predicted a black pot containing a high-reward and was located on the left side of the goal-box. The fine-sandpaper covered tunnel predicted a white pot containing a low-reward and was located on the right side of the goal-box. In the cognitive bias test, the ambiguous tunnel was covered with an intermediate sand texture. The researchers tested whether animals chose the low or high reward pot to assess bias.

Brydges et al. (2011) exposed male rats to standard housing conditions and moved them to standard/unenriched or enriched environments after the first cognitive bias test session (Brydges et al., 2011). Rats that were moved to enriched housings significantly increased positivity-biased choices for the ambiguous tunnel and they were more positivity-biased than rats living in unenriched housings. Strikingly, rats of both groups predominantly foraged in the low-reward location when the ambiguous tunnel was presented, implying that a negativity-biased state was inherent in all rats of standard housing conditions.

Next, Brydges et al. (2012) introduced juvenile stress in female and male rats (Brydges et al., 2012). It was found that female rats learned the task quicker than their male counterparts, but when confronted with the ambiguous probe, females needed more time to reach the gold-box than males. This suggests that males were more impulsive than females when they entered the ambiguous tunnel. Unfortunately, the effect of juvenile stress on the performance of the ambiguous tunnel in male and female rats remained unclear. All rats, however, exposed to juvenile stress made, unexpectedly, more positivity-biased choices than non-stressed animals. Unstressed rats needed more time to choose a pot than stressed subjects during ambiguous trials. A confounding effect might have been that the stressed rats had less mass than the controls. In concordance with the results of Richter et al. (2012), it is possible that stressed animals increased risk taking whilst foraging, in order to obtain food, and therefore took less time to check the new environment, the tunnel covered with the intermediate sandpaper texture, than unstressed rats.

Chaby et al. (2013) introduced unpredictable social and physical stress in adolescent rats (Chaby et al., 2013). They used same methods as Brydges et al. (2011, 2012) used, but the left black pot contained one cheerio (low reward) and the right pot three cheerios (high reward) in this study. On the first day of the cognitive bias test, all stressed rats interpreted the ambiguous trial as negative, while in the non-stressed group, half of the rats interpreted the ambiguous trial as positive and the other half as negative. In subsequent tests, there was overall no difference between stress and non-stressed rats in the interpretation of the ambiguous location, but there was a tendency toward a negative bias in the stressed group. Stressed rats, additionally, tended to have a shorter latency to correct and re-orientate to the correct bowl during discrimination training. Similar to the findings of Brydges et al. (2012) and Richter et al. (2012), animals were more impulsive. That is, they left the shelter quicker than their non-stressed conspecifics in this study (Brydges et al., 2012; Chaby et al., 2013).

In conclusion, non-stressed animals make more positivity-biased choices than stressed animals and enriched housing induces a shift toward positive bias in studies using tactile cues (Brydges et al., 2011, 2012; Chaby et al., 2013).

## Visual

### Cognitive Bias Under Baseline Conditions

Krakenberg et al. (2019) performed a visual task with mice to assess cognitive bias (Krakenberg et al., 2019). Animals were presented with a touch screen framed in plastic so that three windows were accessible. If a bar was presented at the bottom of the central window, mice had to touch the right window to obtain a high reward. If a bar was shown at the top of the central window, mice had to touch the left window to obtain a low reward. The ambiguous cues were three bars appearing in between these reference bars (near-high reward, midpoint, near-low reward). Their responses to choose either left or right window upon appearance of one of the ambiguous cues predicted their cognitive bias toward high (positivity-bias) or low reward (negativity-biased). The author calculated the optimism scores in response to the five bar positions, and found significant differences in the cognitive bias test. The score response to the ambiguous cue of mid-point was lying in between the reference cues. Mice generalized the near-positive cue to the positive and the near-negative cue to the negative cue since the number of responses to choose the window that was associated to the reference cue were similar. It is, nevertheless, possible that mice were not able to visually discriminate between the near-negative and negative cue (or near-positive and positive cue) because of the similar response rates. It was not clear whether there was a cognitive bias when confronted with the visual cue located at the midpoint of the reference cues since the study was probably underpowered and individual data points were not shown. As mentioned previously in the chapter of spatial task, Krakenberg et al. (2019) conducted a second experiment using a paradigm based on actual tunnel distance, in which the width of generalization is narrow. However, the generalization curves obtained by the actual and visual paradigms on distances have not been statistically compared.

## Discussion and Conclusions

**Figure 3:**
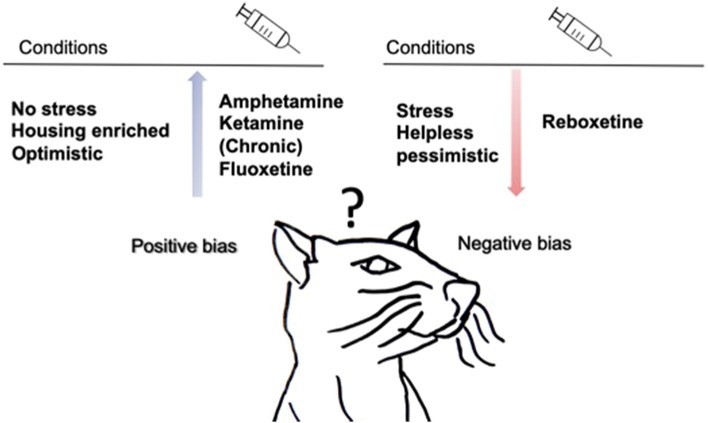
Summary of main findings of animal generalization studies. Drug treatment, traits and environmental conditions influence cognitive bias in rodents. During chronic subcutaneous injection of fluoxetine and after acute injection of amphetamine and ketamine rodents are more positive when confronted with ambiguous cues. Acute intraperitoneal reboxetine injection, make rodents more negativity-biased. Animals that are non-stressed, “positivity-biased” and living in housing enrichment make more positive choices, while negativity-biased, stressed and helpless rodents make fewer positive choices. In addition, an alternative reward replaces the positive bias toward alcohol in rodents.

Figure 3 provides a summary of the main findings. Stressed rodents are less positivity-biased when confronted with an ambiguous probe as they decrease the positive response and increase the negative response in auditory tasks (Papciak et al., 2013; Rygula et al., 2013; Rygula and Popik, 2016; Drozd et al., 2017, 2018). There are indications that stressed animals make less positive choices in tactile tasks (Chaby et al., 2013). Interestingly, negativity-biased rodents are more realistic than positivity-biased rodents since they make more correct choices (Rygula and Popik, 2016). This is consistent with the hypothesis of depressive realism proposing that depressed individuals make more accurate judgments, perceive their performance more accurately, and are able to better assess or recall their performance than non-depressed individuals (Moore and Fresco, 2012). Non-stressed rodents, in contrast, are positively biased compared to stressed animals when using tactile stimuli (Brydges et al., 2011, 2012; Chaby et al., 2013). A meta-analysis in human study from Moore & Fresco support this finding by showing that non-depressed individuals show a significant positive bias (Moore and Fresco, 2012). Positive bias is also observed after environmental enrichment animals shift toward a positive cognitive bias in a tactile task (Brydges et al., 2012). In rats exposed to amphetamine and ketamine a shift in cognitive bias toward positive decision-making was found. The availability of an alternative reward prevents the generalization of ambiguous cues toward alcohol and therefore diminishes alcohol consumption in rodents (Anderson et al., 2013; Ginsburg and Lamb, 2013; Hales et al., 2017). Reboxetine treatment shifts cognitive bias toward negative expectations, whereas chronic fluoxetine administration shifts cognitive bias toward positive expectations (Enkel et al., 2010; Hales et al., 2017).

The risk of bias clearly emphasizes that a golden standard for rodent studies is missing. Rodent characteristics were often not reported concisely, and outcomes were not reliable, thereby hampering the comparison of study results. Overall, the data suggest that cognitive bias was inferred by spatial, tactile, visual and auditory ability. Considering the methodological heterogeneity and risk of bias, the present data should be interpreted with caution.

The auditory and tactile tasks seem to measure negative cognitive bias under negative conditions, and positive cognitive bias under positive conditions, with the auditory task being most extensively validated. The tactile tasks using latency as readout do seem to be associated with potential confounding factors, as conditions like stress can influence latency (e.g., due to decreased body mass and an increased motivation to forage for food) and thereby influence the assessment of cognitive bias. Unlike the auditory and tactile tasks, the visual task has only been validated under baseline conditions, where it remains uncertain whether the animals were actually able to visually discriminate between the near-negative/positive and negative/positive cue. Further studies are thus required determine the visual discrimination abilities of the animals [e.g., by associating the conditioned cues to the same reward (or punishment), and to determine whether the negative cognitive bias turns more negative under negative conditions, and the positive cognitive bias more positive under positive conditions]. Finally, in the spatial tasks it remains questionable whether spatial cues are suitable to measure cognitive bias. Although it was shown that spatial ambiguous locations induce a negative bias if located near the negative location and a positive bias if located near the positive location in mice (Krakenberg et al., 2019), cognitive bias was not confirmed (Burman et al., 2008, 2009; Richter et al., 2012). Further, as the spatial tasks also employ latency as readout for cognitive bias, factors that influence latency can interfere with cognitive bias measurements. Overall, the auditory tasks seem to be most validated and thereby to be most ready for use. However, in case of antidepressant drug effects, specifically the negativity-bias induced by reboxetine, predictive validity seems to be suboptimal. Further studies addressing the predictive validity of the auditory tasks, e.g., using other classes of antidepressant drugs, are warranted for future studies. Despite that other tasks appear less well developed compared to the auditory tasks, we would like to encourage the validation of the tactile, spatial and visual tasks as well. When comparing the effects of comparable conditions across tasks, such as stress exposure, both the auditory and tactile tasks reveal that animals show a shift toward negative cognitive biases, albeit this appears to be more convincing in auditory tasks. However, one study reported that stress exposure in the juvenile period increased positivity-biased interpretation of ambiguous locations in a tactile task at a later age (Brydges et al., 2012). This confound is probably because animals under stress and subsequent weight loss increase their risk of foraging, leading to increased food intake and compensatory growth (Hodos, 1961; Killen et al., 2011). Therefore, evidence of anhedonia-like or less motivation-like behavior as assessed by sucrose assumption is not enough (Enkel et al., 2010; Papciak et al., 2013; Rygula et al., 2013). Additional measures such operant responding under a progressive ratio schedule of reinforcement is important to assess motivation (Hodos, 1961). Furthermore, while stress in auditory tasks was found to increase responses for positive stimuli, stress seemed to decrease overall response latencies in tactile tasks. Whether this depends on the specific experimental conditions of the experiments, or on the tasks, remains to be resolved. Furthermore, it was found that in both the auditory and spatial tasks, helpless animals interpret ambiguous sounds/locations are more negative compared to non-helpless animals. Finally, while animals living under enriched conditions do not alter cognitive bias compared to animals living in unenriched housing in the spatial task (Bethell and Koyama, 2015) this type of housing does shift cognitive bias toward positivity in a tactile task (Brydges et al., 2012). Summarized, the different cognitive bias tasks presumably measure comparable processes, but this is yet uncertain because of the variable findings across tasks and the limited studies applying comparable experimental conditions.

In order to correctly interpret the findings in the present systematic review, the experimental design should be noted, especially the potential cofounds such as the decision strategy of tasks (e.g., go/go or go/no-go style), the type of outcome used (e.g., reward, neutral or aversive), and the physical property of novel probes (only one type of novel probes or several different type of probes). In the articles we collected, six of them used an experimental design with a “go/no-go” strategy (Harding et al., 2004; Burman et al., 2008, 2009; Richter et al., 2012; Kloke et al., 2014; Bethell and Koyama, 2015). In these experiments, subjects learned to distinguish between different types of perceptual information through active approaching behavior and inhibitory behavior. In contrast to the “go/no-go” strategy, the remaining articles used the “go/go” strategy, in which subjects learned to distinguish different types of perceptual information through the same positive actions. Under the experimental design of the “go/no-go” strategy, there are two possible combinations of outcome, “reward/no-reward,” “reward/punishment.” Subjects learned to be rewarded for positive action-type activities and to evade punishment by suppressing action. In the experimental design of the “go/go” strategy, there are also two combinations of outcomes, “reward/reward” and “reward/punishment.” Subjects learned to take positive action to get rewards, and they needed to take positive action to avoid punishment. A disadvantage of “go/no-go” tasks is that they cannot determine whether cognitive bias results from a decrease in positive and/or negative responses. In addition, “no-go” as a response indicator cannot be distinguished from missing responses. Based on this, we believe that the detection of cognitive bias in the “go/go” paradigm is more reliable for measuring emotional valence. In the experiments using a “go/go” strategy with “reward/punishment” combination, rodents learned to avoid being punished proactively when faced with perceptual information associated with aversive outcome (e.g., foot-shock conditioned sound). And rodents proactively get rewards such as sucrose when faced with reward perceptual information (e.g., sucrose conditioned sound) (Enkel et al., 2010; Anderson et al., 2013; Papciak et al., 2013; Rygula et al., 2013; Rygula and Popik, 2016; Drozd et al., 2017, 2018). In “reward/reward” combinations, one reward is more attractive to another reward for rodents (Brydges et al., 2011, 2012; Chaby et al., 2013; Ginsburg and Lamb, 2013; Novak et al., 2016; Hales et al., 2017; Krakenberg et al., 2019). In this paradigm, rodents proactively get rewards by distinguishing the two conditioned information (e.g., two frequencies of sound). The two combinations of “reward/reward” and “reward/punishment” under the “go/go” strategy have their own advantages and disadvantages. In the “reward/reward” combination it is obvious that the detection strength of negative bias is weakened, as all outcomes are all rewarding. Because the subjects have the possibility of being punished (for example, electric shock), the “reward/punishment” combination is a kind of stress derived from the experimental design itself, which would weaken the detection intensity of the research subject in the positive bias direction. Of course, this requires new experiments to prove. In addition, when measuring subjects' judgments to ambiguous cues, the possible adaptation to the cues when repeatedly presented during the ambiguous cue test can lead to a loss of novelty due to learning effects (Doyle et al., 2010). At the same time, the advantage of using a wider range of ambiguity tones is that records of near-positive and negative responses and midpoint tones can be analyzed to investigate the mechanism of generalization function underlying cognitive bias (Ginsburg and Lamb, 2013; Norbury et al., 2018).

There are some limitations to mention. First, we did not contact authors when unclear reporting was detected. Furthermore, at least two independent reviewers should have conducted data extraction and the risk of bias assessment. The concepts used in the search strategy were very abstract. Behavioral concepts, especially their definition in psychology and neuroscience, are not clear or incoherent, which prevents researchers to capture all papers. Finally, we assume that the animals used in the studies reviewed had the sensory capabilities to detect the stimuli used in the auditory, spatial, tactile and visual tasks.

Despite these limitations, we show that the current search strategy yielded studies of interest to develop a comprehensive overview about the current state of research investigating generalization bias in rodents. We argue that measuring cognitive bias in rodents should become a golden standard in behavioral science research in order to assess the affective-cognitive state of animals. In the future, a random effects model for the meta-analysis would be appropriate to extrapolate the overall effect sizes of the different subgroups categorized according to their behavioral task.

## Author Contributions

CG proposed the idea of this manuscript. HN and CG conducted the systematic search of the papers. HN conducted the risk of bias test and wrote the manuscript. CG and JH critically read and edited the manuscript. JH supervised HN and GC.

### Conflict of Interest

The authors declare that the research was conducted in the absence of any commercial or financial relationships that could be construed as a potential conflict of interest.

## Supplementary Material

Table S1 Search strategy in database.

Table S2 Characteristics of included studies. n.a., not applicable; ?, unsure.

Table S3 Risk of bias assessment.
