# Supplementary material for: Cognitive Bias Under Adverse and Rewarding Conditions: A Systematic Review of Rodent Studies
Source: Front Behav Neurosci. 2020 Feb 12;14:14. doi: 10.3389/fnbeh.2020.00014 (PMC7029709; doi:10.3389/fnbeh.2020.00014)
Supplement: Table S1 — Search strategy in database. [file Table_1.pdf]

Table S1: Search strategy in database

**PubMed**
**1. Depression**<br><br>"Depression"[Mesh] OR "Depressive Disorder"[Mesh] OR Depression*[tiab] OR depressive[tiab] OR Melancholia*[tiab] OR stress behaviour*[tiab] OR stress behavior*[tiab] OR<br><br>**2. Addiction**<br><br>"Behavior, Addictive"[Mesh] OR "Substance-Related Disorders"[Mesh] OR addiction[tiab] OR addictive behaviour*[tiab] OR addictive behavior*[tiab] OR Drug Abuse[tiab] OR Drug Dependence[tiab] OR Substance Use Disorder*[tiab] OR Drug Use Disorder*[tiab] OR Substance Abuse*[tiab] OR Substance Dependence[tiab] OR Drug Habituation[tiab] AND<br><br>**3. Generalization 1**<br><br>Attention bias*[tiab] OR Attentional bias*[tiab] OR avoidance learning[tiab] OR cognitive bias*[tiab] OR negative bias*[tiab] OR positive bias*[tiab] OR learning bias*[tiab] OR conditioning[tiab] OR conditioned response*[tiab] OR avoidance behaviour[tiab] OR avoidance behavior[tiab] OR perception bias*[tiab] OR discrimination learning[tiab] OR specific memory[tiab] OR generalized memory[tiab] OR generalised memory[tiab] OR emotional bias*[tiab] OR judgement bias*[tiab] OR memory bias*[tiab] OR interpretation bias*[tiab] OR<br><br>**4. Generalization 2**

"Generalization (Psychology)"[Mesh] OR Generalization OR Generalisation OR Overgeneralization OR Undergeneralization OR Overgeneral OR Overgenerality OR Over-generalization OR Under-generalization OR Over-general OR Over-generality AND

5. animal filter [(1)]

(1) Hooijmans, C. R., Tillema, A., Leenaars, M., & Ritskes-Hoitinga, M. (2010). Enhancing search efficiency by means of a search filter for finding all studies on animal experimentation in PubMed. *Laboratory animals*, *44*(3), 170-175
